# A Dissipative Phenomenon: The Mechanical Model of the Cosmological Axion Influence

**DOI:** 10.3390/e27101036

**Published:** 2025-10-02

**Authors:** Ferenc Márkus, Katalin Gambár

**Affiliations:** 1 Department of Physics, Budapest University of Technology and Economics, Műegyetem rkp. 3, H-1111 Budapest, Hungary; markus.ferenc@ttk.bme.hu; 2 Department of Natural Sciences, Institute of Electrophysics, Kálmán Kandó Faculty of Electrical Engineering, Óbuda University, Tavaszmező u. 17, H-1084 Budapest, Hungary; 3 Department of Natural Sciences, National University of Public Service, Ludovika tér 2, H-1083 Budapest, Hungary

**Keywords:** axion effect, symmetry breaking, frequency shift, cosmology, Lagrangian, Klein-Gordon equations with opposite sign mass terms, dynamic transition

## Abstract

The appearance of a negative mass term in the classical, non-relativistic Klein–Gordon equation deduced from mechanical interactions describes a repulsive interaction. In the case of a traveling wave, this results in an increase in amplitude and a decrease in the wave propagation velocity. Since this leads to dissipation, it is a symmetry-breaking phenomenon. After the repulsive interaction is eliminated, the system evolves towards the original state. Given that the interactions within the system are conservative, it would be assumed that even the original state is restored. The analysis to be presented shows that a wave with a lower angular frequency than the original one is transformed back to a slightly larger amplitude. This description is a suitable model of the axion effect, during which an electromagnetic wave interacts with a repulsive field and becomes of a continuously lower frequency.

## 1. Introducion

In this article, we address the exciting behavior of wave solutions to the Klein–Gordon equations derived from classical mechanical interactions. To achieve our aims, first, we focus on the existing, elaborated mathematical background of Klein–Gordon equations. We show what the consequences of equations with the same origin but differing in the sign of only one term are. After that, we will summarize the consequences of the applied mathematics presented above. In the chosen example, we will assume that a freely propagating wave interacts with a repulsive potential field, and later with a same kind attractive but opposite sign. The calculations show a frequency decrease in the wave which means the dissipation of the original wave. In a sequence of potentials, a redshift effect can be recognized. We point out that this interaction may be similar to that assumed between axions and electromagnetic waves. We briefly summarize the important details for the further calculations below.

### Mathematical Preminilaries: Two Kind of Klein-Gordon Equations

It is a rare mathematical and physical phenomenon that one of the leading terms of a differential equation can have either a positive or negative sign, which is used to describe realistic processes that actually occur. This statement holds for the so-called Klein–Gordon (K-G) equations. The mathematical form of the equation is shown below:

(1) $$\frac{\partial^{2}y}{\partial t^{2}}-c^{2}\frac{\partial^{2}y}{\partial x^{2}}\pm \omega_{0}^{2}y=0.$$

Here, *c* is the propagation velocity without interaction. The $\pm \omega_{0}^{2}y$ (often called mass) term expresses an interaction with different signs that have opposing effects.

The positive sign term pertains to the classical K-G equation [1]. The mechanical model of this motion is a transverse wave traveling on a stretched string, which is also affected by an attractive interaction from the propagation medium, which is proportional to the deflection and perpendicular to the direction of the propagation. Applying a stretching force *F*, a string density ρ, and a cross-section *A* of the string, we can express the free propagation speed as

(2) $$c=\sqrt{\frac{F}{\rho A}}.$$

Furthermore, using the parameter of the attractive perpendicular interaction of $k_{a}$, we obtain

(3) $$\omega_{0}=\sqrt{\frac{k_{a}}{\rho A}}.$$

The energy, *E*, of a particle with momentum *p* and rest mass $m_{0}$ according to the theory of relativity is

(4) $$E^{2}=p^{2}c^{2}+m_{0}^{2}c^{4},$$

where *c* is the speed of light. Introducing the operator calculus,

(5) $$E⟶\frac{\hbar }{i}\frac{\partial }{\partial t}\;\;\;\;\;\mathrm{and}\;\;\;\;\;p⟶\frac{\hbar }{i}\frac{\partial }{\partial x},$$

we obtain a relativistic invariant K-G equation [2]

(6) $$\frac{\partial^{2}\Psi }{\partial t^{2}}-c^{2}\frac{\partial^{2}\Psi }{\partial x^{2}}+\frac{m_{0}^{2}c^{4}}{\hbar^{2}}\Psi =0.$$

Introducing the Compton wavelength related angular frequency,

(7) $$\omega_{0}=\frac{m_{0}c^{2}}{\hbar },$$

we obtain the positive (mass) term relation in Equation (1). All of this shows that the K-G equations with positive mass terms can describe realistic natural processes for both classical and relativistic cases.

Moreover, applying the consecutive series of different sign K-G equations can also be interpreted as a series of interactions in a single phenomenon. The Klein–Gordon equation fulfills these conditions. In this article, we describe a thought experiment that follows the above sign-changing sequence. It is possible that a similar process exists in nature. We will also refer to this.

Steps taken to generalize the Klein–Gordon equations dates back to the work of [3,4]. The appearance of tachyons (faster-than-light particles already proposed in special relativity [5]) and ghost particles in the solutions was interpreted as a kind of problem, but at the same time, it became clear that these possibilities should not necessarily be discarded [6]. In the description of Cherenkov radiation, where the speed of light in a medium can actually be exceeded, tachyons appear as real particles [7]. Although they appear in the description of interactions and play an important role, tachyons in vacuum are not freely propagating particles [8,9].

However, equations with negative mass terms can also be interpreted in classical systems. Even though the description involves the tachyon concept, the problem of exceeding the speed of light does not arise. In a summary article, we showed that in all three classical axiom systems (mechanics, thermodynamis, and electrodynamics), the processes described by the K-G equation with a negative mass term have a physical reality [10]. The processes have similar propagation dynamics, and both show the wave–non-wave dynamics change at the critical point.

In the model under consideration, we study what happens when a freely propagating wave interacts with an attractive and/or repulsive field. For the sake of simplicity, we discuss mechanical waves, but from a technical point of view, this does not limit the model to be applied to other systems. Thus, the described process can be logically paralleled with the interaction (oscillations) of electromagnetic waves with the hypothetical axion field [11,12,13,14,15], or axionic electronic states in topological insulators [16]. This is an interaction that has been considered hypothetical so far in particle astrophysics, but its existence is possible. The model we present here may be similarly realistic. Furthermore, the quest to detect axions as dark-matter particles dates back to the 1980s using microwave resonators [17]. More recently, nitrogen vacancy centers in diamond are proposed to be a suitable candidate to detect axions [18,19,20,21].

We show that a free wave entering a series of repulsive–attractive–repulsive–attractive, etc., interactions undergoes a frequency decrease, which can also be interpreted as red shift-like process.

The article is structured as follows. In Section 2, we explore the relationships between waves propagating freely and under the influence of repulsive and attractive potentials. In Section 3, we explain how frequency and amplitude changes appear in the model. Here we can see the appearance of red shift. In Section 4, we present a series of graphical diagrams calculated for a specific set of parameters showing the changes in the freely propagating wave as it passes through a repulsive and then an attractive interaction. The frequency and amplitude changes predicted in Section 3 are extremely expressive as a function of the magnitude of the potentials. We add a thermodynamic frame calculating the entropy production in a single and an *N* steps transfer in Section 5. Moreover, we express the related entropy formula in the cosmological red shift effect in the case of electrodynamic–axion relation caused damping process. In Section 6, we summarize our results and draw conclusions.

## 2. Three Types of Transversal Wave Propagation—Related Propagation Equations

The subject of the present study is transverse waves generated by various physical effects and their transition into one another. First, we consider a freely propagating wave. Then, the wave reaches a spatial region where it is subject to a perpendicular repulsive effect. The easiest way to imagine this process is the wave being subjected to a centrifugal force resulting from a rotation. This interaction changes both the amplitude and the propagation speed of the wave. We have shown that this change is related to the phenomenon of critical deceleration. Thereafter, the wave enters into a force-free space again. However, this step is not equal to simply eliminating the repulsive effect. We must consider an attractive effect of the same magnitude on the propagating wave, which attempts to restore the original state.

### 2.1. Wave Equation of Free Motion

In the case of free mechanical wave propagation (e.g., along a stretched string), the sum of the potential energy associated with the vertical displacement y(x,t), depending on the space coordinate and time,

(8) $$V=\frac{1}{2}F\int {\left(\frac{\partial y}{\partial x}\right)}^{2}dx,$$

and the kinetic energy associated with the motion is constant, as shown in the following:

(9) $$K=\frac{1}{2}\rho A\int {\left(\frac{\partial y}{\partial t}\right)}^{2}dx.$$

The generated disturbance (signal, vibration state) propagates throughout. Here, *F* denotes the tension force, ρ is the mass density, and *A* is the cross-section of the string.

In the case of conservative interactions, the Lagrange function can usually be given in the form L=K−V. Thus, for a freely propagating wave,

(10) $$L=\int \left[\frac{1}{2}\rho A{\left(\frac{\partial y}{\partial t}\right)}^{2}-\frac{1}{2}F{\left(\frac{\partial y}{\partial x}\right)}^{2}\right]dx.$$

The equation can be written in the usual wave equation form,

(11) $$\frac{1}{c^{2}}\frac{\partial^{2}y}{\partial t^{2}}-\frac{\partial^{2}y}{\partial x^{2}}=0,$$

by the substitution of the speed of signal propagation

(12) $$c=\sqrt{\frac{F}{\rho A}}.$$

### 2.2. Wave Equation with a Repulsive Interaction

When the freely propagating wave reaches the repulsive potential region, the repulsion can be accounted with the potential energy expression

(13) $$V_{cf}=-\frac{1}{2}\int \rho A\omega_{0}^{2}y^{2}\;dx.$$

The $V_{cf}$ term indicates that, in this simple mechanical picture, the repulsion is a the centrifugal effect resulting from the rotation of $\omega_{0}$. Similarly, other more complicated processes can also result in this phenomenon during the interaction of the fields with each other. It is important that the repulsive effect is perpendicular to the direction of propagation. Thus, this stretches the traveling wave in a transverse direction.

To describe the motion that arises in this direction, the repulsive potential in Equation (13) must be subtracted from the Lagrange function in Equation (10). After solving the variational problem, the Euler–Lagrange equation can be derived as an equation of motion

(14) $$\frac{1}{c^{2}}\frac{\partial^{2}y}{\partial t^{2}}-\frac{\partial^{2}y}{\partial x^{2}}-\frac{\omega_{0}^{2}}{c^{2}}y=0,$$

which is a K-G equation with a negative mass term.

In addition to the equation of motion, the Hamilton function is also worth introducing. To do this, first calculate the canonical momentum, *p*, and then use this to calculate the function that gives the total energy

(15) $$p=\frac{\partial L}{\partial (\partial y/\partial t)}=\int \rho A\frac{\partial y}{\partial t}\;dx,$$

(16) $$H=p\frac{\partial y}{\partial t}-L=\int \left[\frac{1}{2}\rho A{\left(\frac{\partial y}{\partial t}\right)}^{2}+\frac{1}{2}F{\left(\frac{\partial y}{\partial x}\right)}^{2}-\frac{1}{2}\rho A\omega_{0}^{2}y^{2}\right]dx.$$

To further investigate the propagation of the wave and its transition through the chain of potentials, we consider a harmonic wave with amplitude $A_{0}$, wavenumber k=2π/λ, here, λ is the wavelength, and angular frequency ω. The propagation is hence described as follows:

(17) $$y\left(x,t\right)=A_{0}\;\sin\left(kx-\omega t\right).$$

It would be meaningless to consider the energy of the entire infinite wave. Instead, we consider a packet of the wave with a finite length of *L*. Then the total energy, *E*, of the wave is given by Equation (17); by substituting in the Hamiltonian in Equation (16), it becomes finite as shown below:

(18) $$E=\frac{1}{4}\rho ALA_{0}^{2}\omega^{2}+\frac{1}{4}FLA_{0}^{2}k^{2}-\frac{1}{4}\rho AL\omega_{0}^{2}A_{0}^{2}.$$

The amplitude $A_{0}$ of the wave is expressed as

(19) $$A_{0}=\sqrt{\frac{4E}{\rho AL}{\left(\omega^{2}+\frac{F}{\rho A}k^{2}-\omega_{0}^{2}\right)}^{-1}}.$$

The solution includes both the free propagation and the repulsive potential cases. This situation is interesting because as soon as the wave reaches the repulsive region, its amplitude increases, while the wavenumber and, with it, the wavelength do not change. Since the angular frequency, ω, decreases to

(20) $$\omega^{\prime }=\omega \left(\lambda ,\omega_{0}\right)=\sqrt{\frac{F}{\rho A}\frac{4\pi^{2}}{\lambda^{2}}-\omega_{0}^{2}},$$

the propagation speed of the wave decreases with increasing $\omega_{0}$. At a certain value of $\omega_{0}$, the wave property changes completely, and a decelerating, vertically expanding dissipative solution appears. This critical slowing-down effect [22,23] is well-known in the dynamic transitions and is a well-identified critical dynamical change: wave ⟶ spreading (⟶ and reaching the limit, towards dissipation). This may be the mechanical model of the axion effect [24]). The continuity of velocity at the transition point ensures that

(21) $$A_{0}\;\omega \left(\lambda ,\omega_{0}=0\right)=A_{amp}^{\prime }\;\omega^{\prime }.$$

With this, the amplitude of the first transition can be expressed as

(22) $$A_{amp}^{\prime }=A_{0}\frac{\sqrt{\frac{F}{\rho A}\frac{4\pi^{2}}{\lambda^{2}}}}{\sqrt{\frac{F}{\rho A}\frac{4\pi^{2}}{\lambda^{2}}-\omega_{0}^{2}}}.$$

In the second transition, we apply an attractive interaction of the same magnitude. The most important question is will the original state be restored?

### 2.3. Wave Equation with an Attractive Interaction

In discussing the attractive interaction, we will assume that the magnitude of the interaction is the same as the repulsion. This is important because, in this way, the effects of the potentials balance each other out energetically. Applying the potential in this case, the K-G equation has the following form:

(23) $$\frac{1}{c^{2}}\frac{\partial^{2}y}{\partial t^{2}}-\frac{\partial^{2}y}{\partial x^{2}}+\frac{\omega_{0}^{2}}{c^{2}}y=0.$$

This is the usual mathematical form. From the dispersion relation, the angular frequency, ω, is

(24) $$\omega \left(\lambda ,\omega_{0}\right)=\sqrt{\frac{F}{\rho A}\frac{4\pi^{2}}{\lambda^{2}}+\omega_{0}^{2}}.$$

The wave arriving in the attractive space must satisfy this.

## 3. Axion Effect on the Frequency: Red Shift Process

Now, in the reverse step, when we turn on restoring, a positive-sign effect appeared instead of the negative mass term. We might think that we simply get back the original amplitude and angular frequency: however, this is not the case. To get the restored amplitude (close to the initial $A_{0}$), taking the reversed mathematical step, we must multiply by the factor $A_{amp}^{\prime }$ in Equation (22) by

(25) $$\frac{\omega^{\prime \prime }}{\sqrt{\frac{F}{\rho A}\frac{4\pi^{2}}{\lambda^{2}}+\omega_{0}^{2}}},$$

assuming that the resulting turning back angular frequency is $\omega^{\prime \prime }$. Thus, the amplitude after the second transition is

(26) $$A_{amp}^{\prime \prime }=A_{0}\frac{\sqrt{\frac{F}{\rho A}\frac{4\pi^{2}}{\lambda^{2}}}\;\omega^{\prime \prime }}{\sqrt{\frac{F}{\rho A}\frac{4\pi^{2}}{\lambda^{2}}-\omega_{0}^{2}}\;\sqrt{\frac{F}{\rho A}\frac{4\pi^{2}}{\lambda^{2}}+\omega_{0}^{2}}}.$$

After the effect of the restoring potential, the amplitude $A_{0}$ does not appear to be the original, but rather somewhat larger. In this section, we assume that the value of $\omega_{0}$ is actually small, because we want to discuss the change in dynamics. On the other hand, because the velocity of passage through equilibrium is constant in each case, the relationship between the products of the amplitude and the frequency is still valid

(27) $$A_{amp}\;\omega \left(\lambda ,\omega_{0}=0\right)=A_{amp}^{\prime \prime }\;\omega^{\prime \prime }.$$

Comparing the previous two equations, we can express the angular frequency $\omega^{\prime \prime }$ after the axion transition

(28) $$\omega^{\prime \prime }=\sqrt[{4}]{{\left(\frac{F}{\rho A}\frac{4\pi^{2}}{\lambda^{2}}\right)}^{2}-\omega_{0}^{4}}=\sqrt[{4}]{\omega^{4}-\omega_{0}^{4}}.$$

The resulting angular frequency is lower than the original. This already means that a kind of red shift phenomenon is occurring.

Originally, we would think that after passing through spaces influenced by conservative potentials of opposite magnitudes, everything would return to its original state. In contrast, we now find that the restored angular frequency is definitely smaller. This phenomenon is dissipation itself. If we look at the phenomenon on a quantum level, we see the appearance of smaller and smaller energy packets at an increased number. At the same time, we have also experienced that a process can be dissipative, despite the fact that only conservative potentials are present. The succession of different fields helps the further energy dissipation.

When the wave propagation returns back to the free case, it achieves the speed $c=\sqrt{F/\rho A}$ again. However, due to the decrease in the angular frequency, a new wavelength should appear. Then we obtain an expression for this wavelength of Λ, which is

(29) $$\Lambda =\frac{2\pi c}{\omega^{\prime \prime }}=\frac{2\pi \sqrt{\frac{F}{\rho A}}}{\sqrt[{4}]{\omega^{4}-\omega_{0}^{4}}}>\lambda .$$

Since the condition $\omega_{0}\ll \omega$ is realistic, it is not necessary to modify the previous calculation, but this fact should be noted. The changed wave number is

(30) $$k^{\prime \prime }=\frac{2\pi }{\Lambda }=\frac{\sqrt[{4}]{\omega^{4}-\omega_{0}^{4}}}{\sqrt{\frac{F}{\rho A}}}<k.$$

If we consider a quantum particle with wavenumber $k^{\prime \prime }$, then the energy loss due to spreading $\Delta \varepsilon =\hbar ck-\hbar ck^{\prime \prime }$ is clearly visible. This is calculated when the $\omega_{0}\ll \omega$ approximation holds

(31) $$\Delta \varepsilon =4\hbar \omega_{0},$$

which is independent of the above-described repulsive–attractive transition, and the original wave angular frequency ω. Assuming that such a process operates in nature, this means that the axion space is full with these loss-originated quantum packets. These are present as low-energy, homogeneous background energies, presumably with very low interaction affinity.

Finally, the question arises, what happens if an attractive effect occurs first, and then a repulsive one of the same magnitude? Going through the previous line of thought, it can be seen that in this case, the amplitude will decrease

(32) $$A_{amp}^{\prime \prime \prime }=A_{0}\frac{\sqrt{\frac{F}{\rho A}\frac{4\pi^{2}}{\lambda^{2}}}}{\sqrt{\frac{F}{\rho A}\frac{4\pi^{2}}{\lambda^{2}}+\omega_{0}^{2}}},$$

and the angular frequency

(33) $$\omega^{\prime \prime \prime }=\omega \left(\lambda ,\omega_{0}\right)=\sqrt{\frac{F}{\rho A}\frac{4\pi^{2}}{\lambda^{2}}+\omega_{0}^{2}},$$

will increase in the first transition. In the second step, the repulsive effect takes effect, resulting in an angular frequency of $\omega^{\prime \prime \prime \prime }$, so the present multiplication factor is

(34) $$\frac{\omega^{\prime \prime \prime \prime }}{\sqrt{\frac{F}{\rho A}\frac{4\pi^{2}}{\lambda^{2}}-\omega_{0}^{2}}},$$

by which the resulted amplitude is

(35) $$A_{amp}^{\prime \prime \prime \prime }=A_{0}\frac{\sqrt{\frac{F}{\rho A}\frac{4\pi^{2}}{\lambda^{2}}}\;\omega^{\prime \prime \prime \prime }}{\sqrt{\frac{F}{\rho A}\frac{4\pi^{2}}{\lambda^{2}}+\omega_{0}^{2}}\;\sqrt{\frac{F}{\rho A}\frac{4\pi^{2}}{\lambda^{2}}-\omega_{0}^{2}}}.$$

It is still the case that

(36) $$A_{amp}\;\omega \left(\lambda ,\omega_{0}=0\right)=A_{amp}^{\prime \prime \prime \prime }\;\omega^{\prime \prime \prime \prime },$$

by which we obtain the previous angular frequency decrease relation

(37) $$\omega^{\prime \prime \prime \prime }=\sqrt[{4}]{\omega^{4}-\omega_{0}^{4}}.$$

This relationship is the same as the result obtained in Equation (28). This means that regardless of the order of passing through the potentials, the decrease in angular frequency is the same. The sequence of successive repulsive–attractive potentials leads to a continuous angular frequency decrease, which can be given by the mathematical form

(38) $$\omega^{\left(n\right)}=\sqrt[{4}]{\omega^{4}-n\omega_{0}^{4}}$$

after the *n*th pair of events. The phenomenon described in this way can rightly be called a red shift.

In the presented approximation, we used the principles of mechanics. However, wave propagation and energetic aspects can be transferred to electromagnetic signal propagation without any problems. Therefore, the appearance of this red shift can be interpreted similarly for the case of electromagnetic waves.

## 4. Graphical Discussion

The mathematical results of the previous section are detailed in figures. The parameters of the freely propagating wave in Equation (17) are amplitude $A_{0}=1$, wavelength λ=2π, so wave number k=1, the angular frequency is ω=1 since the tension force is F=1, the density is ρ=1, and the cross-section is A=1. The mechanism of potential-influenced wave propagation is presented for five values of $\omega_{0}=0.8$; 0.85; 0.9; 0.95; 0.97. These represents the repulsive and later the attractive interactions. All of the parameters are in arbitrary units. We would like to emphasize that the physical condition is still that $\omega_{0}$ is much smaller than ω. In order to make the transitions visible in the diagrams to be created, we choose values close to ω. But we do this only for the sake of visualization.

In all five cases, the freely propagating wave arriving from the left at four different time instants (shown by the color scales: t=−0.2; 0; 0.2; 0.4) reaches the repulsive region marked by the value $\omega_{0}$ at the location with coordinate x=−2π. The amplitude increase calculated by Equation (22) can be clearly identified. At the same time, it is also seen that the wavelength has not changed. Since according to Equation (21), the amplitude change is associated with the change in the angular frequency, the propagation speed decreases. This cannot be read from the figure.

At the point with coordinate x=2π, the repulsive interaction is replaced by the attractive interaction. It is clear from the figures that the amplitude has decreased, but it has not returned to the original $A_{0}$. The angular frequency decreases according to Equation (26). The retraction potential makes the wave propagate freely, so its speed is again *c*. This, in turn, means that the wave number decreases and the wavelength increases. In the series of figures, this can be seen that with increasing $\omega_{0}$ values, there are fewer and fewer waves in the (2π,8π) interval.

**Figure 1:**
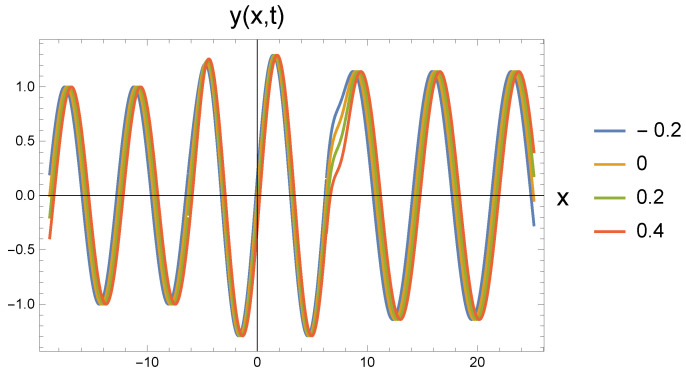
Wave transitions between force-free and repulsive potential fields, and reversibly, by an attractive interaction, the repulsive and force-free fields. The repulsive field is characterized by $\omega_{0}=0.8$ in dimensionless unit.

Figure 1 shows the basic process for the case $\omega_{0}=0.8$. When the repulsive effect appears, the amplitude increases, while for the attractive effect, it decreases, but becomes larger than the value corresponding to the initial state. The transitions are smooth. The amplitudes quickly settle to the stationary value. Along with the amplitude change, the outgoing wavelength becomes larger than the incoming one. Although this is not very noticeable in the figure, it becomes more significant for increasing $\omega_{0}$ parameters.

**Figure 2:**
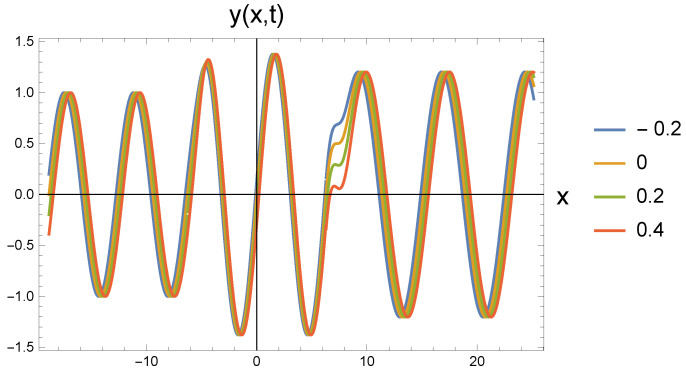
Wave transitions between force-free and repulsive potential fields, and reversily, by an attractive interaction, the repulsive and force-free fields. The repulsive field is characterized by $\omega_{0}=0.85$ in dimensionless unit.

By increasing the value of $\omega_{0}$, both the amplitude and wavelength changes become more pronounced, as can be seen in Figure 2. The change in wavelength can be easily read from the point where the wave exits the figure when compared to Figure 1. The roughness of the second transition depends on the phase relations of the change in the wavelength of the outgoing wave at the transition point.

**Figure 3:**
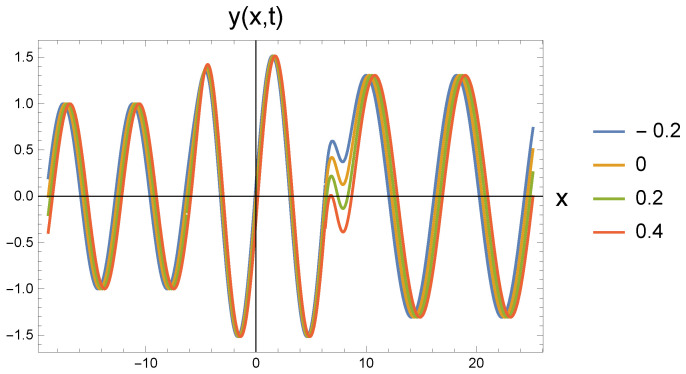
Wave transitions between force-free and repulsive potential fields, and reversibly, by an attractive interaction, the repulsive and force-free fields. The repulsive field is characterized by $\omega_{0}=0.9$ in dimensionless unit.

**Figure 4:**
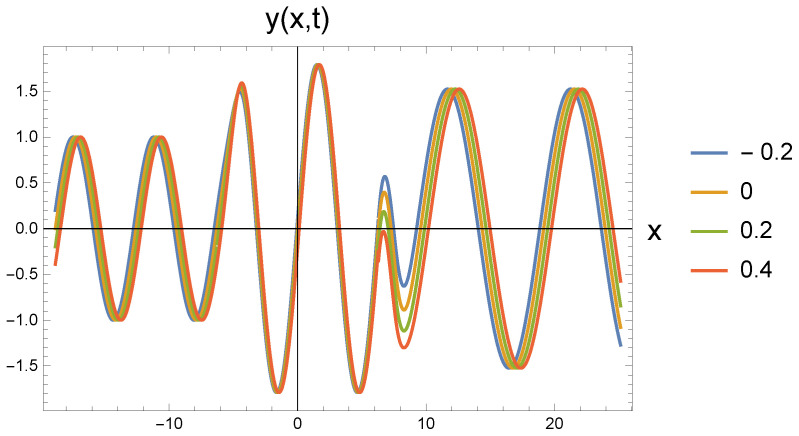
Wave transitions between force-free and repulsive potential fields, and reversibly, by an attractive interaction, the repulsive and force-free fields. The repulsive field is characterized by $\omega_{0}=0.95$ in dimensionless unit.

By further increasing $\omega_{0}$ to 0.9 and 0.95, the statements made above are still valid. However, it is worth observing how much the curves for the middle repulsive potential in the related Figure 3 and Figure 4 slide into each other, while they spread apart in the case of the attractive differential effect. It is clear that in the case of repulsion, the wave gets closer and closer to the critical deceleration, i.e., the wave does not travel in the *x*-direction, but starts to spread out in the perpendicular *y*-direction. Due to the decreasing velocity in the *x*-direction, the waves that are further apart in time push each other. On the other hand, when the attractive effect takes effect, the wavelength increases, which can be read directly from both of the figures. It is also noticeable how much more the wavelength stretches in Figure 4 than in Figure 3.

**Figure 5:**
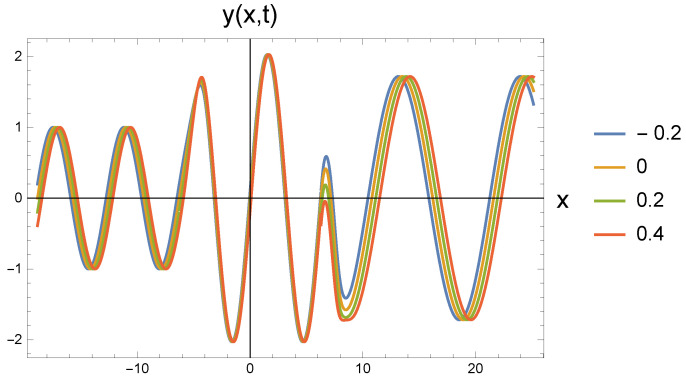
Wave transitions between force-free and repulsive potential fields, and reversibly, by an attractive interaction, the repulsive and force-free fields. The repulsive field is characterized by $\omega_{0}=0.97$ in dimensionless unit.

The transitions shown in Figure 5 are for $\omega_{0}=0.97$. This is very close to the critical point of $\omega_{0}=1$. Given that the repulsive transition is only one wavelength long, the amplitude barely reaches its maximum. It is clear that for a multiple wavelength transition the stationary state is reached. Without this, the transients dominate the figure. The increased amplitude and wavelength in the final state are very clearly visible.

Based on these previous results, it is not difficult to imagine that in the case of the attractive–repulsive sequence, the amplitude first decreases, and then increases. To such an extent that it becomes larger than that of the initial incoming wave. The wavelength increases, and with it the frequency decreases. This is important because in a sequence of interactions, attractive–repulsive–attractive–repulsive, etc., there is no distinguished first event.

## 5. Entropy Production of the Red Shift in the Cosmological Microwave Background Radiation

The above calculations are perfectly applicable to the case of waves propagating in space over long periods in time. Perhaps the best example of this is the interpretation of the red shift of the cosmic microwave background radiation as a consequence of an electromagnetic–axion interaction. The thermodynamic embedding that can be connected to the phenomenon can help in understanding fundamental natural processes. The equivalent thermodynamic temperature of radiation can be expressed by the most common wavelength or frequency of electromagnetic radiation. To achieve our goals, let us first consider the spectral energy density of black body radiation according to wavelength

(39) $$u_{\lambda }\left(\lambda ,T\right)=\frac{8\pi hc}{\lambda^{5}}\frac{1}{e^{\left(hc/\lambda k_{B}T\right)}-1}.$$

It reaches a maximum value at

(40) $$\frac{\partial u_{\lambda }}{\partial \lambda }=0.$$

After derivation, the obtained reduced equation is

(41) $$x-5(1-e^{-x})=0,$$

where we applied the substitution in the simplification

(42) $$x=\frac{hc}{\lambda k_{B}T}.$$

Solving Equation (41), the obtained root is x=4.965; thus, the equivalent temperature of an ω main value radiation is

(43) $$T=\frac{\hbar \omega }{4.965k_{B}}.$$

At this point, we can introduce the thermodynamic framework. The angular frequency decrease in a single repulsion–attraction potential transition from Equation (37) is

(44) $$\Delta \omega =\omega -\sqrt[{4}]{\omega^{4}-\omega_{0}^{4}}\approx \frac{\omega_{0}^{4}}{4\omega^{3}},\;\;\;\;\;\;\;\;\;\;\omega_{0}\ll \omega .$$

This gives an entropy increase at a temperature radiation corresponding to the above-defined temperature *T*

(45) $$\Delta S=\frac{\hbar \Delta \omega }{T}=1.24k_{B}{\left(\frac{\omega_{0}}{\omega }\right)}^{4}=1.24k_{B}\frac{1}{s^{4}}\;\;\;\;\;\;\;\;\;\;s=\frac{\omega }{\omega_{0}},$$

which can be conveniently expressed with the most common angular frequency occurring in the radiation according to Equation (43).

The wave with the reduced angular frequency travels on the next potential passage, so in the following steps, we calculate with the remaining (temperature-related main) angular frequency ω−Δω, ω−2Δω, …, and ω−(N−1)Δω in the *N*th transmission. We can assume that the above statements apply to all waves, so even though we are talking about one wave, the relationships are valid to the entire system. This means that the system is constantly cooling due to the loss, so the rate of entropy production also changes. The related entropy increase in the *n*th step is

(46) $$\Delta S_{n}=\frac{\hbar \Delta \omega }{T}=1.24k_{B}{\left(\frac{\omega_{0}}{\omega -\left(n-1\right)\omega_{0}^{4}/4\omega^{3}}\right)}^{4}.$$

The total entropy production can be expressed by the sum over all steps

(47) $$S_{N}=\sum_{n=1}^{N}1.24k_{B}{\left(\frac{4s^{3}}{4s^{4}+1-n}\right)}^{4}.$$

This sum can be approximated by the integral

(48) $$S_{N}=\int_{1}^{N}1.24k_{B}{\left(\frac{4s^{3}}{4s^{4}+1-n}\right)}^{4}dn=105.8k_{B}{\left[\frac{s^{12}}{{\left(4s^{4}+1-n\right)}^{3}}\right]}_{1}^{N}.$$

Since 1<<N<s, we can approximate the above result

(49) $$S_{N}=105.8k_{B}\left(\frac{s^{12}}{{\left(4s^{4}+1-N\right)}^{3}}-\frac{1}{64}\right)\approx 1.24k_{B}\frac{N}{s^{4}}=1.24k_{B}N{\left(\frac{\omega_{0}}{\omega }\right)}^{4}.$$

The largest-observed red shift is that of the cosmic microwave background radiation [25]. Its numerical value is

(50) $$z=\frac{\omega_{\mathrm{emitted}}-\omega_{\mathrm{measured}}}{\omega_{\mathrm{measured}}}\approx 1100.$$

Applying it in our case, we can identify the parameters as

(51) $$\omega_{\mathrm{emitted}}=\omega ,$$

and

(52) $$\omega_{\mathrm{measured}}=\omega -N\Delta \omega =\omega -N\frac{\omega_{0}^{4}}{4\omega^{3}}.$$

Taking these equations, we can express the unknown product $N\omega_{0}$ by the measured *z* value

(53) $$N\frac{\omega_{0}^{4}}{4\omega^{3}}=\frac{z}{z+1}\omega ,\;\;\;\;\;\;\;i.e.,\;\;\;\;\;\;\;N\frac{\omega_{0}^{4}}{\omega^{4}}=4\frac{z}{z+1}.$$

Now, we can express the entropy production of a red shift photon during its life to present

(54) $$S_{N}=4.965k_{B}\frac{z}{z+1}\approx 6.84\cdot 10^{-23}J/K.$$

The realization we propose is only one of the possibilities leading to red shift. However, this approach may contribute to our understanding of the thermodynamics of cosmic processes [26].

## 6. Conclusions

We have shown that in a complex process sequence, the propagation does not necessarily return to its original state. The three successive processes examined in order: first, starting from a free wave propagation ⟶ second, wave propagation under a repulsive potential interaction ⟶ third, wave propagation under an attractive interaction equal to the magnitude of the repulsive potential. Both the attractive and repulsive potentials are conservative. Nevertheless, the amplitude and frequency of the outgoing freely propagating wave change. This is the result even if we apply the attractive potential first and then the repulsive potential. This fact highlights that there is a much more general principle behind this series of phenomena. As the amplitude increases, the (angular) frequency decreases, so we can observe the dissipation of energy. The vibration shifts towards lower frequencies, so if we look at it quantumly, we can talk about smaller energy packets. A larger amplitude means more particles. In fact, we perceive the decrease of the initial energy packet as dissipation.

Such an interpretation of the phenomenon may even partially explain the red shift, or at least there may be such a contribution. Light arriving from a distance can participate in such an attractive–repulsive, but mutually balancing, process series several times, or even continuously. As a result, the frequency of the light continuously decreases in small steps. The number of events is directly proportional to the distance so that a greater distance results in a greater frequency decrease. The phenomenon, of course, only produces measurable results on an astronomical scale.

## Data Availability

The original contributions presented in this study are included in the article. Further inquiries can be directed to the corresponding author.
